# Supervised Digital Neuropsychological Tests for Cognitive Decline in Older Adults: Usability and Clinical Validity Study

**DOI:** 10.2196/17963

**Published:** 2020-09-21

**Authors:** Francesca Lunardini, Matteo Luperto, Marta Romeo, Nicola Basilico, Katia Daniele, Domenico Azzolino, Sarah Damanti, Carlo Abbate, Daniela Mari, Matteo Cesari, Nunzio Alberto Borghese, Simona Ferrante

**Affiliations:** 1 Nearlab Department of Electronics, Information and Bioengineering Politecnico di Milano Milano Italy; 2 AISLab Department of Computer Science University of Milano Milano Italy; 3 School of Computer Science The University of Manchester Manchester United Kingdom; 4 Geriatric Unit Fondazione IRCCS Ca’ Granda Ospedale Maggiore Policlinico Milano Italy; 5 Department of Nutritional Sciences University of Milano Milano Italy; 6 Department of Clinical and Community Sciences University of Milano Milano Italy

**Keywords:** aging, Bells Test, computerized testing, dementia, early diagnosis, eHealth, mild cognitive impairment, neuropsychological assessment, Trail Making Test

## Abstract

**Background:**

Dementia is a major and growing health problem, and early diagnosis is key to its management.

**Objective:**

With the ultimate goal of providing a monitoring tool that could be used to support the screening for cognitive decline, this study aims to develop a supervised, digitized version of 2 neuropsychological tests: Trail Making Test and Bells Test. The system consists of a web app that implements a tablet-based version of the tests and consists of an innovative vocal assistant that acts as the virtual supervisor for the execution of the test. A replay functionality is added to allow inspection of the user’s performance after test completion.

**Methods:**

To deploy the system in a nonsupervised environment, extensive functional testing of the platform was conducted, together with a validation of the tablet-based tests. Such validation had the two-fold aim of evaluating system usability and acceptance and investigating the concurrent validity of computerized assessment compared with the corresponding paper-and-pencil counterparts.

**Results:**

The results obtained from 83 older adults showed high system acceptance, despite the patients’ low familiarity with technology. The system software was successfully validated. A concurrent validation of the system reported good ability of the digitized tests to retain the same predictive power of the corresponding paper-based tests.

**Conclusions:**

Altogether, the positive results pave the way for the deployment of the system to a nonsupervised environment, thus representing a potential efficacious and ecological solution to support clinicians in the identification of early signs of cognitive decline.

## Introduction

### Background

In the near future, the exponential growth of the number of people 65 years or older is expected to have an impact on health care systems because of the physical and cognitive decline typically associated with the aging process. In terms of cognitive decline, population aging goes hand in hand with the rapid increase in the number of people with dementia and the related increase in public costs. Indeed, as reported by the World Health Organization, the total estimated worldwide cost of dementia in 2010 reached US $604 billion, of which approximately one-third was spent in Western Europe [1].

The transient step between physiological aging and dementia is known as mild cognitive impairment (MCI); MCI is a condition characterized by cognitive weakening that is not yet producing a clinically significant effect on daily activities but can be detected through clinical examinations or formal cognitive tests [2,3]. Currently, there is no effective treatment for dementia; however, nonclinical interventions such as cognitive training started in the MCI stage [4] can delay the onset of dementia and extend the duration of independent living [5]. Therefore, early diagnosis is crucial.

Currently, neuropsychological assessment represents an important tool for the diagnosis of dementia and MCI. It consists of a set of multi-item rating scales and batteries of brief cognitive tests that evaluate the different cognitive functions and is administered by a specialist in controlled environments, typically hospitals or clinical facilities [6]. The controlled environment setting might easily delay the diagnosis [7] for 2 main reasons: (1) the long waiting times of outpatient facilities [8] and (2) the fact that individuals are often examined after the manifestation of symptoms and when serious concerns are raised by their referents. In addition to this delayed diagnosis, a recent meta-analysis [9] showed that the proportion of undetected dementia is above 60%.

In this framework, the American Psychological Association recognized the importance of computerized testing [10] as an essential part of the screening procedures in the nearest future. Indeed, computerized assessment has potential advantages over the widely used paper-and-pencil testing, including cost and time efficiency, accurate recording of responses, automatic extraction of quantitative features related to test performance, computation of additional fine indicators, and comparison of the patient’s outcome between different sessions over time. Owing to the pervasiveness of contemporary computing technologies, computerized testing can be a solution to counter delayed diagnosis of dementia, eventually allowing the adoption of proper preventive measures; such tools can be used in clinical facilities to increase the effectiveness of medical services or be deployed in home environments to detect possible cognitive impairment earlier than sporadic medical visits.

### Prior Work

Although initial attempts to introduce computerized versions of the classic paper-and-pencil neuropsychological tests have been reported [11-16], the achievement of feasible, effective, and ecological computerized testing is hampered by 3 main factors: (1) the lack of normative data to support tool validity, (2) technology use anxiety of older adults, and (3) the challenging implementation of test supervision. Concerning the first challenge, Zygouris and Tsolaki [17] pointed out that in most cases data supporting the concurrent validity of computerized testing with formal paper-and-pencil tests are largely incomplete or inconclusive. Against this backdrop, 2 computerized test batteries—the CNS Vital Signs (CNSVS; CNS Vital Signs, LLC) [18] and the Cambridge Neuropsychological Test Automated Battery (CAN-TAB; Cambridge Cognition Ltd) [19]—were particularly successful in providing normative databases. The CNSVS [18] is self-administered and implements a number of heterogeneous tests, including finger tapping, verbal and visual memory, test of shifting attention, digit symbol coding, Stroop test, and continuous performance test. The CAN-TAB [19] is administered by a trained technician and includes various tests that assess visual memory, executive function, attention, semantic and verbal memory, decision making, response control, and social cognition that can be combined into different batteries.

For the second challenge, the reduced familiarity with technology that characterizes older adults can affect both their performance and willingness to undergo computerized testing. Werner and Korczyn [20] conducted interviews to examine factors associated with the expressed willingness to use computerized systems to diagnose dementia and reported a strong effect of technology anxiety, particularly for participants with lower socioeconomic status and for female users. In this framework, NeuroTrax Mindstreams (NeuroTrax Corporation) [16] was reported to be particularly user-friendly [21]. Mindstreams implements digitized adaptations of tests designed to study different domains. Tests are performed with the support of an examiner who is needed throughout the testing process and include assessing verbal and nonverbal memory, visual-spatial skills, verbal fluency, information processing, attention, executive function, and motor skills. In a study by Fillit et al [22], the battery was rated as easy to use by most older adults, even by those with significant cognitive impairment.

Concerning the third challenge, despite the great achievements of state-of-the-art computerized testing systems, automatizing test supervision remains to be a crucial problem. Usually, computerized testing is either performed under the supervision of an expert professional (as requested for the CAN-TAB and Mindstreams batteries) or self-administered (as for the CNSVS). In the first case, home-deployment of computerized testing is hindered by the need for a professional, whereas in the latter case, the absence of supervision to guarantee and guide proper test execution in uncontrolled environments may affect test results [17].

### Objectives

In this study, we developed a platform that provides a computerized version of 2 neuropsychological tests commonly used to assess dementia and MCI, the Trail Making Test (TMT) [23] and the Bells Test [24]. The goal was to go one step further to present state-of-the-art systems and provide a tool that could effectively support the diagnosis of cognitive decline. Our system provides test supervision through an intelligent vocal assistant (VA) embedded in the platform and introduces a replay functionality to allow remote inspection of the user’s performance after test completion. Such innovative functionalities potentially allow the delivery of the system directly to the user’s home or for usage in clinical facilities without strict supervision to support current medical services. To adopt the system in nonsupervised environments, extensive functional testing of the platform was performed, together with a clinical validation of the computerized version of the tests. The validation had the two-fold aim of evaluating the system’s usability and acceptance and investigating the concurrent validity of the computerized assessment compared with the corresponding paper-and-pencil tests in identifying clinical cognitive decline. This paper presents the results obtained from a study on 83 older adults.

## Methods

### Design Approach and Definition of Requirements

Batteries of neuropsychological tests are usually administered by a neuropsychologist at clinical premises. The neuropsychologist explains the test to the user, supervises test execution (possibly notifying and correcting the user in case of errors), and assigns the score after test completion. The level of supervision is strictly dependent on each test protocol.

To design the supervised digitized neuropsychological tests, the principles of the design thinking process were adopted [25]. First, brainstorming sessions with clinical experts (neuropsychologists and geriatricians) and technical partners were organized to identify user and functional needs. The following requirements were obtained:

- Test supervision should mimic the one offered by the neuropsychologist during the paper-based version of the test. To this aim, events requiring supervision should be promptly and consistently recognized to trigger the related intervention.
- Test raw data and indicators should be stored and accessible for evaluation by authorized experts. Pseudonymization of data should be provided.
- A replay functionality—capable of recording a test session, reproducing it, and highlighting relevant events—should be provided to allow clinicians to remotely inspect the test after its execution.
- The digitized version of the test should be conveyed by standard consumer technology.
- The digitized version of the test should be intuitive and allow easy interactions with the user (as tests are designed to be performed by patients with cognitive decline).
- Test scores should not depend on any speech-based interaction with the user, which should aid in the execution of the actual test.

The prototyping process consisted of different testing iterations to refine the requirements and, consequently, system development. Clinicians and target users were involved during these early stages of design. The first testing iterations consisted of showing the software to a team of geriatricians and neuropsychologists. Once the software was collectively approved, some tests on older adults were performed to check the software behavior with the target users and to gather additional feedback for usability improvement. The feedback from 4 subjects recruited from a senior association was leveraged to improve the system and fine-tune the interface and the interfacing modalities (eg, implementation of palm rejection, inclusion of additional written indications to facilitate test comprehension and execution, implementation of specific software improvements to minimize errors in the automatic scoring). After these refinements, testing was conducted on a large population of users.

### Test Selection

On the basis of the identified functional requirement and the experience of clinicians, the TMT including its 2 conditions, Part A (TMT-A) and Part B (TMT-B), and the Bells Test were chosen.

The TMT was chosen as it is a well-established predictor of cognitive functional abilities in both healthy older adults and patients with MCI [23]. In the TMT-A, subjects are asked to draw a line connecting, in sequential order, 25 target numbers presented on an A4 paper sheet (portrait orientation). The TMT-B requires subjects to connect, in sequential and alternate order, 13 target numbers and 12 target letters (ie, 1, A, 2, B...N, and 13). Both tests must be executed as quickly as possible, possibly without lifting the pen from the paper. If patients make an error, the examiner reorients them to the last correct target. The test ends when the correct sequence is completed with the last target, and the main outcome is the time of completion. The 2 versions of the test allow the examiner to assess different cognitive domains: psychomotor speed and tracking for the TMT-A and processing speed, mental flexibility, and executive functions for the TMT-B. Furthermore, to better isolate the cognitive processes associated with the TMT-B performance, it is common practice to subtract the time required to complete the TMT-A from the time required to complete the TMT-B, thus deriving a new parameter (TMT-BA) [26].

The Bells Test was selected to investigate attentional functions through a visual search task. In the version proposed by Gauthier et al [24], subjects are asked to find and mark 35 targets (black-ink drawings of bells), presented on an A4 paper sheet (landscape orientation), among 280 distractors. If the subject stops before all the bells are encircled, the examiner gives one incitement to verify that all the targets have been found. After this incitement, the test ends when subjects stop their searching activity. The main outcome of the test is the number of targets correctly identified.

### Supervised Computerized Neuropsychological Tests

#### System Architecture

**Figure 1:**
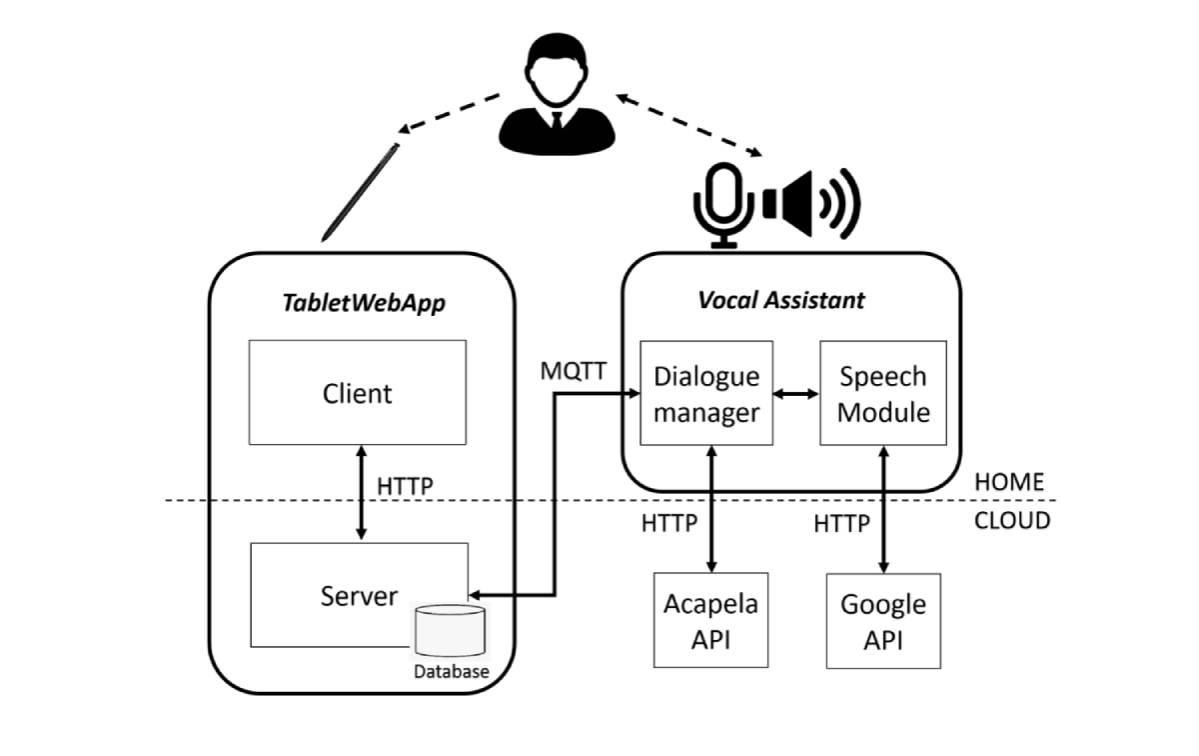
System architecture. API: application programming interface; HTTP: Hypertext Transfer Protocol; MQTT: Message Queue Telemetry Transport.

The supervised computerized neuropsychological tests platform presents a hierarchical architecture (Figure 1) based on the integration of 2 main components: an app running on a tablet that implements the digitized tests (TabletWebApp) and an intelligent VA that provides the supervision required to conduct the test autonomously in an uncontrolled environment. The web app, TabletWebApp, displays the tests on a tablet, processes in real time the trace executed by the user, recognizes relevant events such as users’ errors, and interacts with the VA during test execution. The VA, owing to its interactions with the TabletWebApp during test execution, interactively explains the test to the user, supervises test progression, and provides adequate feedback to the user through speech interventions. The integration between the 2 main components is enabled by a bidirectional communication channel following the Message Queue Telemetry Transport (MQTT) protocol, a publish or subscribe transport layer built on top of TCP/IP (Transmission Control Protocol and Internet Protocol) widely used for internet of things applications. The messages sent and received via this channel allow the TabletWebApp and VA to exchange information about the present state of the test, so that consistent speech interventions can be triggered.

The TabletWebApp comprises a server component that resides in the cloud and a client component embodied in the tablet (Figure 1). A Samsung Galaxy Tab A6 10.1 with S Pen (115 mm long stylus, with a squared section of 6.5 mm×5 mm) was used, thus making it the most similar solution to the paper-and-pencil approach. The client displays, at a frame rate of 30 Hz, the current view of the test (as received from the server) along with the trajectory of the stylus to provide the user with the sensation of drawing on the screen. It also logs the current stylus position and communicates it to the server in real time. The client features a user front end based on HTML5 and JavaScript with graphical user interfaces (GUIs). The client interfaces have been developed to work on any touch device to be adaptable to the users’ needs; the test canvas is displayed on a full screen to avoid distraction. The server component is based on Node.js; it keeps track of the present state of the test and provides the app’s view via http to the tablet client. To secure communication, the encrypted http secure protocol is leveraged between the client and server. All raw data (along with the indicators and parameters are presented in the Digitized TMT and Bells Test section) are stored server-side inside a NoSQL (Not only Structured Query Language) database.

As for the VA, a voice user interface was chosen to meet the requirement of an easy and intuitive interaction with the user. Previous work [27] on senior users showed that a voice-based interaction increases the overall engagement with the device, compared with the alternative of operating a GUI. The VA interacts with users through speech, adopting a 2-phase implementation: (1) speech recognition and (2) speech synthesis. The VA includes a speech module and a dialogue manager. The speech module encompasses a speech recognition service that exploits the speech-to-text Google Cloud Platform Speech Application Programming Interface (API). The dialogue manager implements a high-level logic for speech interaction, supervises speech by running a Finite-state Machine that tracks the present state of the dialogue engaged with the user, manages the speech synthesis based on Acapela Voice as a Service, and manages the interaction with TabletWebApp via MQTT.

The dialogue manager calls the speech module whenever an utterance from the user is needed for the correct flow of the interaction. The speech module is responsible for opening the microphone to listen to the user’s utterances, sending the recorded utterance to the Google Cloud Platform for recognition, and analyzing the text returned from the Google Speech API. To provide the most natural interaction, the VA has been designed to recognize different utterances that provide the same semantic meaning. For this reason, the inputs detected by the microphone and processed by the Google API are further analyzed to look for relevant keywords. The speech from the user is sent to the Google Speech API which returns a text with the transcription of the user’s speech. In this text, the speech module looks for relevant keywords that are organized in sets of different input dictionaries so that different utterances with the same semantic meaning are clustered in a single semantic input (eg, “Yes, I understood,” “I have understood,” and “I understand” are associated to the same semantic input “understood”). Once a relevant keyword is found, it is considered as the user’s answer and is communicated to the dialogue manager. The dialogue manager then selects the next correct utterance of the VA from a set of output dictionaries and reproduces the associated audio file obtained through the Acapela API. Each output dictionary is appropriate for the specific state of the dialogue manager, which is changed after receiving the answer from the speech module. Once a transition between the 2 states of the dialogue manager is executed, a message is sent to the TabletWebApp, which properly reacts to work in parallel with the VA. The state transitions of the dialogue manager can also be activated by the TabletWebApp, which automatically recognizes the user’s actions and sends a message to trigger proper transition.

As described by its architecture, the platform is distributed across a local client, and a server is deployed in the cloud. Consequently, all the described functionalities constantly require a reliable internet connection.

#### Digitized TMT and Bells Test

**Figure 2:**
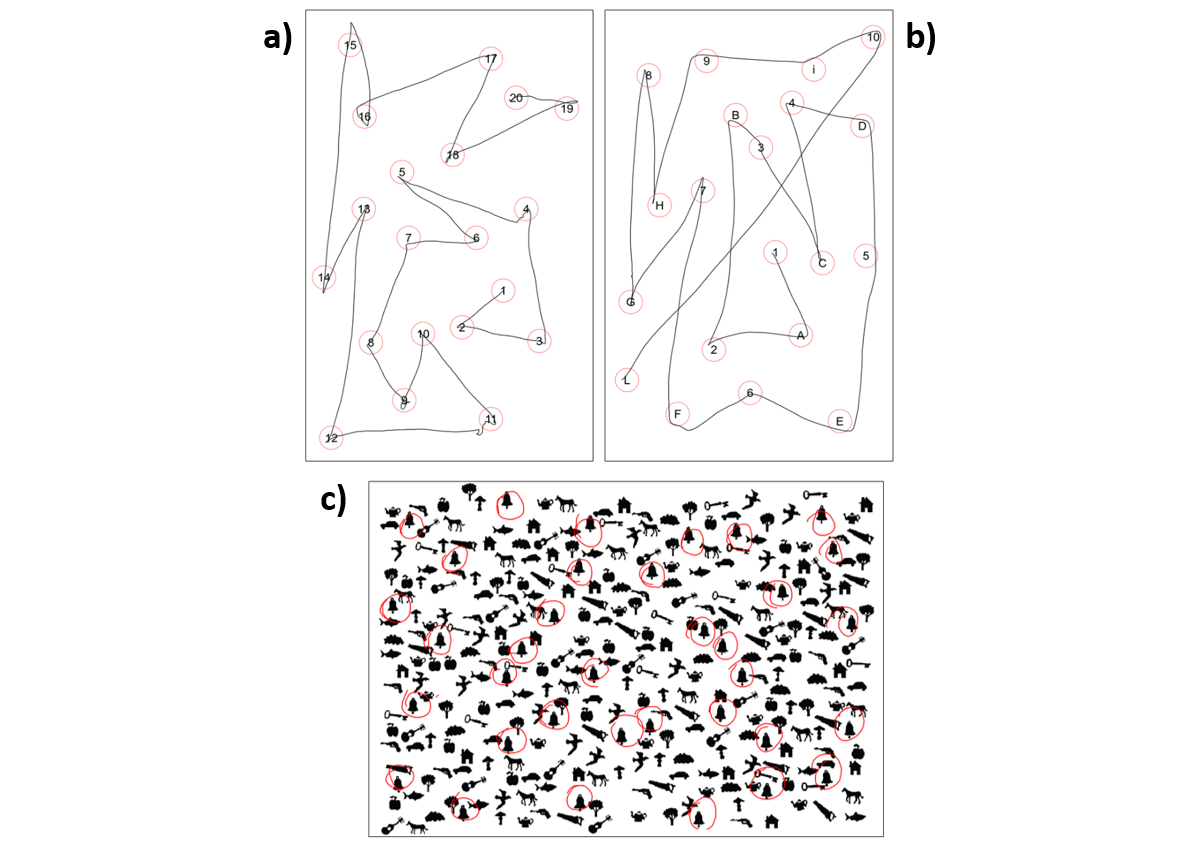
Layout of the digitized tests: (a) Trail Making Test (TMT)-Part A, (b) TMT-B, and (c) Bells Test.

The digitized TMTs were designed to be structurally similar to the paper-based version proposed by Giovagnoli et al [28]; however, given the reduced dimensions of the tablet (14.8 cm × 21 cm) compared with the A4 paper sheet, the number of targets was decreased to 20 for both versions (TMT-A: 1-20; TMT-B [Italian version]: 1A-10L; and TMT-B [English version]: 1A-10J; Figure 2) [13]. For both TMT-A and TMT-B, the first and last targets were indicated by a written sign. In the digitized TMT, a target is considered reached when the stylus trace hits the target active area, which corresponds to the circle shown on the screen. To increase the robustness of the platform, the active area of the last target is increased by 20% when it is next to be reached according to the correct order. This choice was made because, as noticed from pretesting on potential users (see the *Design Approach and Definition of Requirements* section), it may occur that at the end of the test, the pen trace approaches the final target without really entering the circle. The software logs the events (target hit), analyzes the connection order, and classifies each reached target as a correct target (respecting the order), a repeated target (previously hit in the sequence), or an error (violating the order). The TMT starts automatically when the first target is entered and ends automatically when the last target is reached after being connected in the correct order with all previous ones or after a 5-min timeout.

The layout of the digitized Bells Test, on the other hand, was identical to that proposed by Vallar et al (35 targets and 280 distractors) [29]; however, all graphical elements were scaled to fit the tablet size (Figure 2). Rather than keeping the symbol size constant and reducing the number of symbols compared with the paper-based version, we opted for this solution to maintain the original configuration of the Bells Test with the characteristic distribution and ratio between targets and distractors. Moreover, pretesting on potential users (see the *Design Approach and Definition of Requirements* section) reported that the symbol size on the tablet was not considered problematic for test execution. In the digitized Bells Test, a target is considered as found when a collision occurs between the stylus trace and the target active area. The target’s active area here is a circle centered in the symbol centroid, with an area that exceeds the symbol surface by 20%. The software logs the events (target found), analyzes the symbol marked, and classifies it as a correct target (bell) or as an error. The Bells Test ends when all the bells have been correctly identified, when the user believes to have found all the bells and declares it, or after a 5-min timeout.

For both tests, the test layout data (target coordinates) and the collected raw data (stylus coordinates at each time instant) are stored in a cloud database, together with a log of events that occurred during test execution. Each detected event is described in the log by the following fields: target ID, timestamp, 2-dimensional pen coordinates (if present), event type (target-TMT: if the correct target was connected; target-Bells: if a bell was correctly identified; error-TMT: if a target was connected in the wrong order; error-Bells: if a symbol different than a bell was identified; and repetition-TMT: if a target already correctly connected in the sequence was repeated).

The stored data, anonymized in compliance with users’ privacy, are used with a two-fold purpose: (1) computing test indicators and (2) providing a remote replay functionality.

To compute test indicators for the 2 tests, the following evaluation indicators are chosen:

- TMT:
  - Time: time required to complete the test (main outcome of the test).
  - Targets: total number of target-TMT events.
  - Errors: number of error-TMT events.
  - Errors/targets the number of error-TMT events divided by the number of target-TMT events.
  - ΔT: the average time between 2 successive target-TMT events.
  - ΔT_n: the time between 2 successive target-TMT events, averaged over the last 21−n target-TMT events (with 1≤n≤20).

- Bells:
  - Targets: total number of target-Bells events (main outcome of the test).
  - ΔT: the average time between 2 successive target-Bells events.
  - ΔT_n: the time between 2 successive target-Bells events, averaged over the last 36−n target-Bells events (with 1≤n≤35).
  - NIDmin: The number of inversions in direction (NID) was computed as a measure of the regularity of the patient’s scanning strategy. The *scanning* trace of the Bells Test execution was calculated by connecting the coordinates of the first 25 target bells in the order of detection and then decomposed into horizontal (x) and vertical (y) components. The x- and y-scanning traces were smoothed (moving average over a span of 3 elements). For each of the 2 directions, the NID was computed as the number of zero-crossings of the derivative of the smoothed trace. As the scanning strategy can be organized either in a vertical or horizontal pattern, the smallest NID over the 2 directions was retained.
  - 1Bell: the location of the first target-Bells event, expressed in terms of subarea. The canvas of the test is divided into 9 rectangular subareas of equal sizes (3 rows [1, 2, and 3]×3 columns [A, B, and C]).

Regarding the second aim, the test data are used to provide a remote replay functionality, which reproduces the test and highlights all the events that had been detected by the software. The aim of the replay is to provide clinicians and caregivers with the possibility of watching a deferred session of the test through any web browser.

#### Test Progression

**Figure 3:**
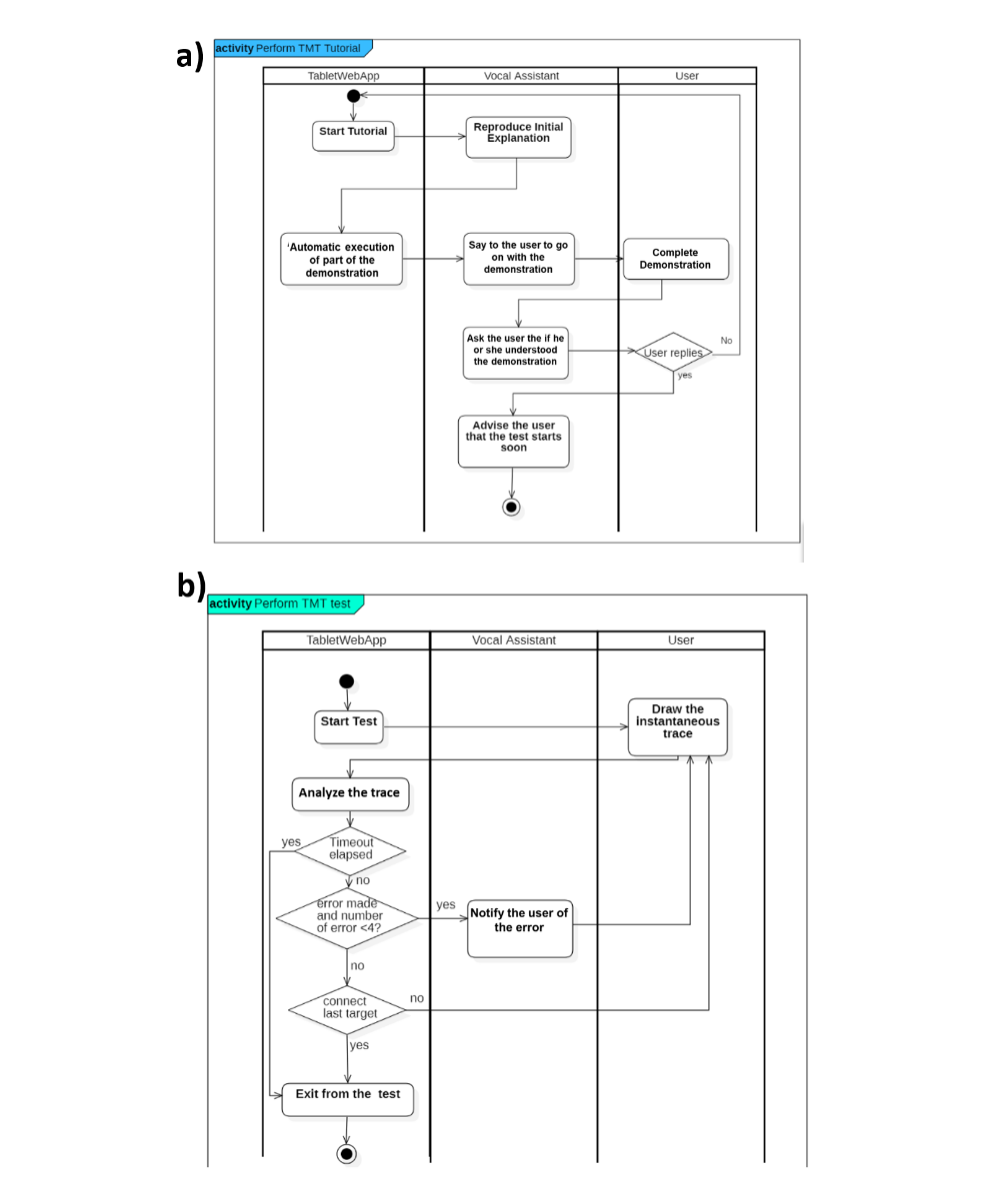
Unified Modeling Language activity diagram for the (a) Trail Making Test tutorial and (b) test execution.

The session begins when the user starts the test app by tapping its icon on the tablet’s home screen. First, a short explanation is presented by the VA. Once the user acknowledges the comprehension of the explanation, the tutorial of the digitized test starts. Such a tutorial aims to replicate the one conducted by the neuropsychologist during the paper-based test and consists of a short example of the test: a small subset of targets is presented to the user, who hears the VA explaining the test while the app performs the initial part of the demonstration by highlighting the first targets; at the end, the VA asks the user to practice with the remaining ones. The tutorial flowchart for the TMT is shown in Figure 3. Once the tutorial is over, the actual test starts. For the TMT, as soon as the user fails to connect targets in the right order, an error is detected by the tablet app, and an error message is shown on the tablet screen. At this point, virtual supervision is triggered: the VA notifies the user that an error has been made and that test execution should be resumed from the last target correctly identified. As in the paper-based test, the user receives notifications for a maximum of 3 errors. The high-level activity diagram of the TMT execution is shown in Figure 3. During the Bells Test, supervision is implemented under the following 2 conditions: (1) if no additional bells are identified for more than 45 seconds, the VA asks the users if they believe to have found all the targets, and the test is either resumed or terminated based on the users’ answer, or (2) if the users declare to have finished the test, the VA recognizes the command and the test is terminated.

TMT-A and TMT-B are conducted in a row, and, at the end of both tests, users are asked for their availability to take the Bells Test. At the end of the whole assessment, the VA reproduces a general accomplishment notification to the user (eg, “Thank you for taking part in this test”). Such a general remark is provided regardless of the actual results of the tests, as requested by clinical partners following the paper-and-pencil evaluation**.**

### System Testing and Validation

In this section, we report a series of tests conducted on the devised system. First, functional testing was executed to verify the robustness of the entire platform (see the *System Architecture Functional Testing* section). In addition, validity and usability of the tablet-based neuropsychological testing were evaluated for potential users (see the *Clinical Validation of the Digitized Tests section*).

#### System Architecture Functional Testing

The 2 components of the proposed framework, namely the TabletWebApp and the VA, were extensively evaluated in terms of robustness and functionality both as stand-alone components and as an integrated system.

##### Functional Evaluation of VAs

Supervision over test execution is performed by a series of speech-based interactions between the VA and the user. Test supervision mimics the one performed by clinicians at clinical premises and consists of a series of sentences with a very simple structure. Speech recognition is of paramount importance as issues related to the understanding of the users' answers may undermine the effectiveness and usability of the entire platform, resulting in misuse of the system. The correct identification of the semantic meaning of the user’s utterances and the correct interaction flow were assessed in a controlled setting (ie, university laboratory) on adult subjects. No constraint on the age of the users was imposed as the subject’s age should not affect the speech recognition performance of the Google Cloud Platform Speech APIs. Subjects were instructed to interact with the system without any specific constraint to test the robustness of recognizing natural language variants. Possible noise sources that could undermine the performance of VA speech recognition were simulated (eg, background noise coming from a television and people talking in the background). Overall, 200 tests were performed.

##### Functional Evaluation of the Integrated System

To test the communication and integration of the 2 main components (TabletWebApp and VA), a set of 15 experimental runs with all possible branches of the interaction between the user, VA, and the TabletWebApp were performed. From the data collected, a detailed analysis of the workflow steps was performed by computing the following metrics: (1) passed steps, that is, steps successfully completed with their expected outcome; (2) incomplete steps, that is, steps not completed because of adverse events/errors, whose possible occurrence was expected and consequently managed by the system (eg, MQTT disconnection); (3) blocked steps, that is, steps not started because of incomplete previous steps; and (4) failed steps, that is, steps resulting in unexpected outcomes, which were not managed by the system.

#### Clinical Validation of the Digitized Tests

Testing the validity and usability of the tablet-based TMT and Bells Test is necessary before a possible deployment of the full system guided by a VA in an unsupervised environment. For this reason, the digitized tests were performed on a group of older adults in clinical premises. During the clinical validation of the digitized tests, the experimental sessions were conducted under the supervision of a clinician specifically trained to use the tablet app.

##### Participants

With the aim of improving the early assessment of cognitive decline, this study addresses older adults who do not have high cognitive impairment. For this reason, the following inclusion criteria were defined: (1) aged ≥65 years and (2) a Mini-Mental State Examination score ≥20. Participants were recruited by neuropsychologists of the geriatric unit of the Foundation IRCCS (Istituti di Ricovero e Cura a Carattere Scientifico) Ca' Granda Ospedale Maggiore Policlinico (Milan, Italy) from among the patients undergoing a neuropsychological visit that included the administration of a battery of standard neuropsychological tests, including the original paper-based TMT and Bells Test.

The ethical board of the Foundation IRCCS Ca' Granda Ospedale Maggiore Policlinico approved the study protocol (October 12, 2017; n°642_2017bis).

##### Experimental Protocol

To reduce the effect of possible facilitation of the computerized test execution, each participant started the battery of standard neuropsychological tests with the paper-and-pencil TMT and Bells Test. On the basis of the outcome of the visit, the neuropsychologist provided a diagnosis and the subject was categorized into 1 of the following 3 groups: *normal*, *MCI*, or *dementia*.

The administration of the digitized tests took place after the completion of the clinical neuropsychological assessment so that the computerized tests were performed about 2 hours after the paper-based tests. Participants were seated at a table with the tablet flat in front of them. In case of poor eyesight, participants were instructed to wear their glasses. The digitized neuropsychological tests were performed under the supervision of a trained clinician in the following order: (1) TMT-A, (2) TMT-B, and (3) Bells Test. Before each digitized test, subjects were provided with an oral explanation of the test and were allowed to practice the tutorial on the tablet. Each digitized test was started only after the participant had shown a proper understanding of the execution mechanism.

After the completion of the computerized neuropsychological tests, subjects were asked to fill out a questionnaire. The questionnaire consisted of 2 parts:

- A series of 7 questions were related to the ease of use of the digital platform, the clarity of the provided explanations and the graphical interface, the overall satisfaction, and the user’s familiarity with technology. The following questions were rated on a 3-point Likert scale:
  - I believe the test explanation and tutorial were clear.
  - I have found the graphical interface of the TMT to be simple and intuitive.
  - I have found the graphical interface of the Bells Test to be simple and intuitive.
  - I am satisfied with the overall experience.
  The following were yes or no questions:
  - Have you ever used a computer?
  - Have you ever used a tablet?
  - Have you ever used a smartphone?

- The Italian version of the System Usability Scale (SUS) questionnaire [30].

Once the experimental session was completed, a neuropsychologist (who was not present during the execution of the digitized tests) remotely watched users’ performances and scored the computerized tests observed through the replay functionality.

##### Data Analysis and Statistics

Data analysis was conducted with 4 main aims to (1) test system acceptance and usability, (2) test system validity, (3) study and compare the discriminative power of the paper-based and digitized test outcomes, and (4) study the digital-specific indicators in the different diagnostic groups.

###### System Acceptance and Usability

For each diagnosis group separately (dementia, MCI, normal) a frequency analysis (to understand how often each answer was chosen within the same group) was conducted on the answers to the items of the questionnaires was carried out. As for the SUS, the original scores were converted into a 0 to 100 range by following the guidelines provided in a study by Brooke [30]. An SUS score above 68 was considered above average.

###### System Validity

System validity was assessed by comparing the scores assigned to the digitized test by the software with both the performance assessed by a neuropsychologist through the replay functionality (see the *Digitized Test Versus Replay* section) and the score obtained in the paper-based counterpart (see the *Digitized Test Versus Paper-Based Test* section).

- Digitized Test Versus Replay
  In the TMT, the sporadic occurrence of the error-TMT events resulted in zero-clustered data that were not well suited for a correlation analysis [31]; therefore, the sensitivity, specificity, and accuracy of the computerized tests were computed according to the following definitions: true-positive is the number of error-TMT events correctly identified; true-negative is the number of target-TMT events correctly identified; false-positive is the number of events erroneously identified as error-TMT; and false-negative is the number of error-TMT events not identified.
  For the Bells Test, the Spearman correlation between the outcome of the digitized tests automatically computed by the TabletWebApp (targets) and the score assigned by a clinician to the execution of the same test inspected through the replay functionality was analyzed.
- Digitized Test Versus Paper-Based Test
  Concurrent validity of the digitized neuropsychological tests with regard to the paper-based tests was tested by running a Spearman correlation between the primary outcomes (time for TMT-A, TMT-B, and TMT-BA and targets for Bells Test) of the digitized and paper-based tests. In cases of a weak correlation, we investigated whether 1 of the 2 versions of the test systematically returned a higher number of targets through a Kruskal-Wallis test.

###### Discriminative Power of Neuropsychological Test Outcomes

For both paper-based and digitized versions, we studied the possible differences in test outcomes (time for TMT-A, TMT-B, and TMT-BA and targets for Bells Test) based on the subjects’ neuropsychological diagnoses (*normal*, *MCI*, and *dementia*). The Kruskal-Wallis test with a Bonferroni adjustment for pairwise comparison was performed.

###### Discriminative Power of Novel Indicators Derived From the Digitized Tests

To study the ability of the digital-specific indicators to distinguish the 3 diagnostic groups (*normal*, *MCI*, and *dementia*), the Kruskal-Wallis test (with a Bonferroni adjustment for pairwise comparison) was performed on the following indicators: errors/targets ratio for TMT-A and TMT-B; ΔT for TMT-A, TMT-B, and Bells Test; and NIDmin for Bells Test. The trend of ΔT_n (TMT and Bells Test) and 1Bell (Bells Test) indicators according to the neuropsychological diagnosis was also studied, together with the scanning strategy during the Bells Test.

The significance level was set at 5% for all tests. Nonparametric statistical analyses were performed after verifying that the data were not normally distributed (Lilliefors test). The analyses were performed using RStudio version 1.0.143 (RStudio Inc).

## Results

An example of the execution of the supervised neuropsychological test is provided in Multimedia Appendix 1 (the video presents the supervised execution of the 3 neuropsychological tests by an adult).

### System Architecture Functional Testing

The correct identification of the semantic meaning of the users’ utterances and the correct interaction flow were assessed on 3 subjects (2 women aged 25 and 26 years and 1 man aged 35 years).

The speech recognition functionality of the VA, evaluated over 200 tests, resulted in the detection of 92.5% (185/200) of the answers. The undetected answers can be ascribed to 2 main reasons: (1) excessive noise in the trials performed with added background noise and (2) wrong timing (eg, the user replied too soon, when the VA was not yet listening). A total of 91.9% (170/185) of detected answers were correctly recognized in the first attempt. In the remaining 8.1% (15/185) of cases, the VA automatically asked the user to repeat the answer. Only 1 repetition per answer was requested by the VA before it was able to correctly recognize the answer.

The workflow of the integrated system was tested in all possible execution branches, and no issues were encountered (100% (255/255) passed steps and 0% incomplete, blocked, and failed steps). During these runs, the system required the repetition of only 3 vocal inputs provided by the user. Given this result, the functional evaluation of the system architecture was considered successful. A demonstration of the interaction between the VA and TabletWebApp has also been proposed and tested during a series of live sessions [32,33].

### Clinical Validation of the Digitized Tests

This study presents the results from 83 recruited patients. From the clinical neuropsychological visits, 27% (22/83) patients were categorized as *normal*, 59% (49/83) as *MCI*, and 15% as (12/83) *dementia* (Table 1). The age of the participants in the 3 diagnostic groups was not statistically different (*P*=.28). Among the patients selected by the neuropsychologist after the clinical visit, 6% (5/89) refused to take part in the study, whereas 1 patient who was recruited decided not to continue with the experimental trial because of an eye disease. All participants gave written informed consent for participation and authorization for the use of protected health information as reported in the ethical submission documentation.

**Table 1:** Characteristics of the participants (overall and divided into diagnosis groups).

Diagnosis groups		Normal	Mild cognitive impairment	Dementia	Total
Population, n		22	49	12	83
**Age (years)**					
	Mean (SD)	76.2 (4.2)	78.0 (5.4)	78.6 (4.0)	77.6 (4.9)
	Range	69-84	65-93	71-82	65-93
Mini-Mental State Examination score, mean (SD)		28.9 (1.1)	27.5 (2.2)	22.8 (1.9)	27.1 (2.7)
Education (years), mean (SD)		12.4 (4.4)	10.3 (4.6)	8.8 (5.1)	10.6 (4.9)
**Sex, n (%)**					
	Female	16 (73)	23 (47)	8 (67)	47 (57)
	Male	6 (27)	26 (53)	4 (33)	36 (43)

#### System Acceptance and Usability

The results obtained for system acceptance and usability are reported in Tables 2-4. The satisfaction questionnaire showed strong positive results. Indeed, most users (95% (21/22) normal, 91% (45/49) MCI, and 83% (10/12) dementia) were pleased with the overall experience. More specifically, the graphical interfaces of both the TMT (90.5% (20/22) normal, 83% (41/49) MCI, and 100% (12/12) dementia) and the Bells Test (81% (18/22) normal, 86% (42/49) MCI, and 83% (10/12) dementia) were considered simple and intuitive. In addition, the tutorial at the beginning of each test was found to be easy to understand by most users (100% (22/22) normal, 91% (45/49) MCI, and 100% (12/12) dementia). These data are particularly important if we consider that most users had limited familiarity with touch technology, as found from the questionnaires. Although some participants were able to use a computer (57% (13/22) normal, 37% (18/49) MCI, and 33% (4/12) dementia), few of them had used a smartphone (38% (8/22) normal, 17% (8/49) MCI, and 50% (6/12) dementia), and even fewer participants were acquainted with tablet technology (9.5% (2/22) normal, 6% (3/49) MCI, and 0% (0/12) dementia).

**Table 2:** System acceptance results from questions rated on a 3-point Likert scale.

General questions	Answers, n (%)				Diagnosis
	Agree	Neutral	Disagree		
**I believe the test explanation and tutorial were clear**					
	22 (100)	—^a^	—	Normal	
	45 (91)	1 (3)	3 (6)	MCI^b^	
	12 (100)	—	—	Dementia	
**I have found the graphical interface of the TMT^c^ to be simple and intuitive**					
	20 (90.5)	—	2 (9.5)	Normal	
	41 (83)	5 (11)	3 (6)	MCI	
	12 (100)	—	—	Dementia	
**I have found the graphical interface of the Bells Test to be simple and intuitive**					
	18 (81)	—	4 (19)	Normal	
	42 (86)	1 (3)	6 (11)	MCI	
	10 (83)	—	2 (17)	Dementia	
**I am satisfied with the overall experience**					
	21 (95)	1 (5)	—	Normal	
	45 (91)	4 (9)	—	MCI	
	10 (83)	—	2 (17)	Dementia	

^a^Indicates 0%.

^b^MCI: mild cognitive impairment.

^c^TMT: Trail Making Test.

**Table 3:** System acceptance results from yes or no questions.

General questions	Answers, n (%)		Diagnosis	
	Yes	No		
**Have you ever used a smartphone?**				
	8 (38)	14 (62)		Normal
	8 (17)	41 (83)		MCI^a^
	6 (50)	6 (50)		Dementia
**Have you ever used a tablet?**				
	2 (9.5)	20 (90.5)		Normal
	3 (6)	46 (94)		MCI
	—^b^	12 (100)		Dementia
**Have you ever used a computer?**				
	13 (57)	9 (43)		Normal
	18 (37)	31 (63)		MCI
	4 (33)	8 (67)		Dementia

^a^MCI: mild cognitive impairment.

^b^Indicates 0%.

**Table 4:** System usability results from the System Usability Scale questionnaires.

Diagnosis	SUS^a^ score, mean (SD)	Participants with a positive score ≥68 (%), n (%)
Normal	82.0 (16)	19 (86)
Mild cognitive impairment	76.0 (17)	38 (77)
Dementia	77.5 (12)	10 (83)

^a^SUS: System Usability Scale.

In addition, for the SUS scores, positive results in terms of system usability emerged for all 3 groups: the average SUS score was 82 (SD 16) for normal, 76 (SD 17) for MCI, and 77.5 (SD 12) for dementia groups. Overall, 86% (19/22), 77% (38/49), and 83% (10/12) of the participants reported a positive SUS score (≥68) for the normal, MCI, and dementia groups, respectively.

#### System Validity

##### Digitized Test Versus Replay

For the TMT-A, the automatically identified error-TMT and target-TMT events by the software were compared with the ground truth that could be obtained by leveraging the replay functionality, reporting a sensitivity of 85.71%, specificity of 99.45%, and accuracy of 99.39%. For the TMT-B, a sensitivity of 72.16%, specificity of 98.39%, and accuracy of 96.84% was attained.

As for the Bells Test, the target indicator was strongly correlated with the one reported by the clinician through the replay (ρ=0.96; *P*<.001).

##### Digitized Test Versus Paper-Based Test

For both TMT-A and TMT-B, the completion time obtained in the digitized versions was strongly correlated with the completion time of the paper-based tests (TMT-A: ρ=0.68, *P*<.001; TMT-B: ρ=0.78, *P*<.001). Similarly, a significant strong correlation between the score of the digitized and the paper-based versions was found for TMT-BA (ρ=0.70; *P*<.001). On the other hand, the correlation between the targets in the digitized and paper-based versions of the Bells Test was significant but weak (ρ=0.39, *P*<.001). However, the Kruskal-Wallis test did not reveal any systematic difference between the 2 versions of the test as 45% (54/83) of the subjects achieved a better performance in the digitized test, whereas 44% (53/83) of them did better in the paper-based version, with a similar average number of targets identified in the 2 modalities of the test (digitized: mean 32.01 [SD 2.75]; paper-based: mean 31.80 [SD 3.74]).

#### Discriminative Power of the Neuropsychological Test Outcomes

**Figure 4:**
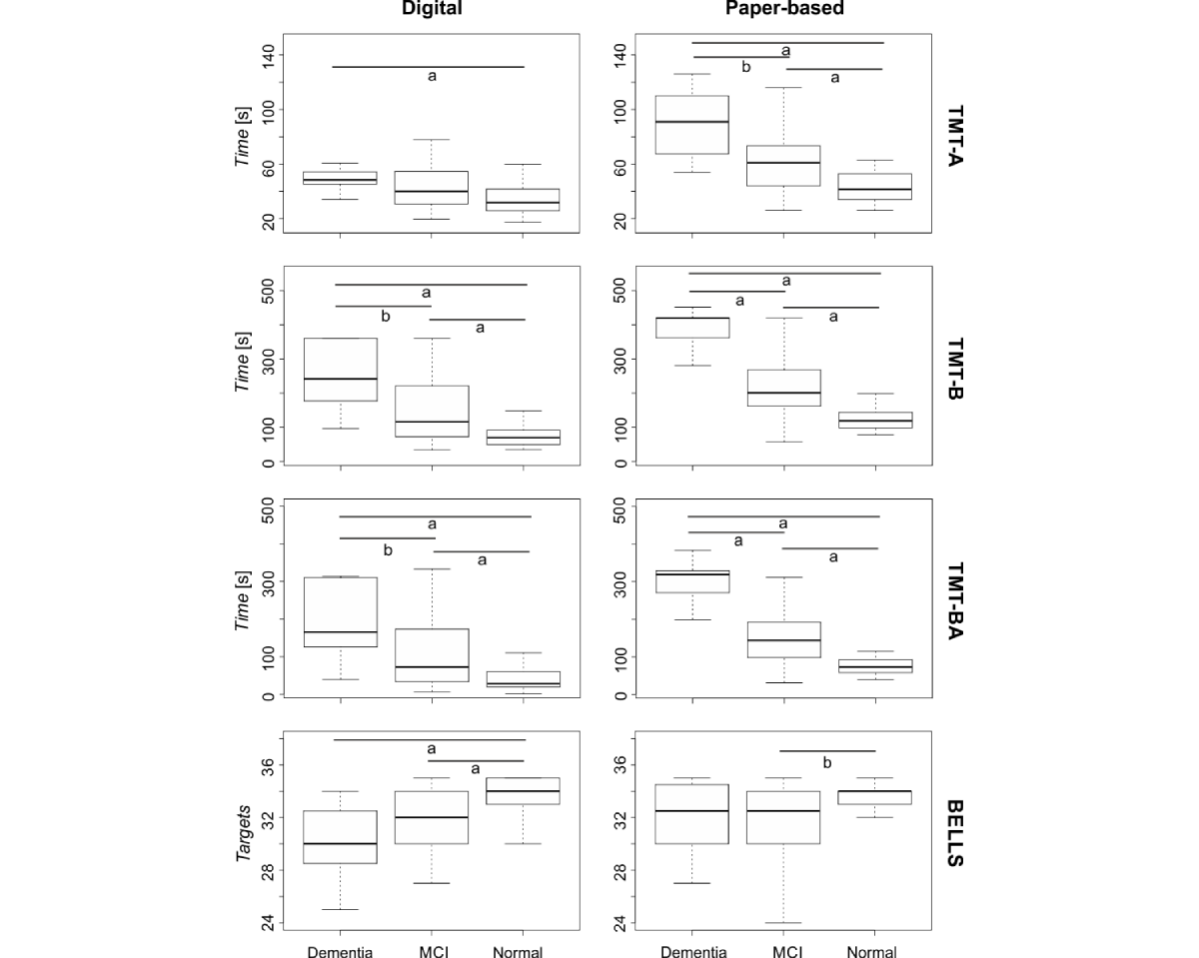
Differences in test scores based on the participant’s neuropsychological diagnosis. The boxplots represent, for the 3 neuropsychological diagnosis groups (normal, mild cognitive impairment, and dementia), the main outcome of the tests (time for Trail Making Test [TMT]-Part A, TMT-B, and TMT-BA and targets for Bells Test) as median and IQRs. Asterisks indicate significance from pairwise comparison (a indicates 1% significance and b indicates 5% significance ).

For the TMT-A, the completion time obtained in the paper-based version was significantly different according to the diagnosis *(X^2^*_2_*=*22.6; *P*<.001), with values increasing as the diagnosis worsened. The posthoc analysis highlighted significant differences between all the groups (normal vs dementia: *P*<.001; normal vs MCI: *P*=.003; and MCI vs dementia: *P*=.02). As can be observed in Figure 4, the same significant trend was found for the digitized test (*X^2^*_2_=12.2; *P*=.002). However, the posthoc analysis showed a significant difference only between normal and dementia groups (*P*=.002) and an almost significant *P* value when comparing normal and MCI (*P*=.06) groups.

For the TMT-B, a significant effect of diagnosis on time emerged for both the paper-based (*X*^2^_2_=36.2; *P*<.001) and digitized (*X^2^*_2_=21.9; *P*<.001) tests, with values increasing as the diagnosis worsened. For both versions of the test, the posthoc analysis reported significant differences between all the groups (paper-based test: normal vs dementia *P*<.001, normal vs MCI *P*<.001, and MCI vs dementia *P*<.001; digitized test: normal vs dementia *P*<.001, normal vs MCI *P*=.004, and MCI vs dementia *P*=.02).

The TMT-BA showed similar results. Indeed, a significant effect of diagnosis emerged for both the paper-based (*X^2^*_2_=33.3; *P*<.001) and digitized *(X^2^*_2_*=*17.1; *P*<.001) versions, with more time required by patients with more severe symptoms. The posthoc analysis revealed significant differences between all the groups (paper-based test: normal vs dementia *P*<.001, normal vs MCI *P*<.001, and MCI vs dementia *P*<.001; digitized test: normal vs dementia *P*<.001, normal vs MCI *P*=.01, and MCI vs dementia *P*=.05).

Finally, for the Bells Test, the diagnosis had a significant effect on the targets of the paper-based *(X^2^*_2_*=*7.2; *P*=.03) and the digitized *(X^2^*_2_*=*18.9; *P*<.001) versions. However, as can be seen in Figure 4, only for the digitized version, we can observe a clear trend with fewer targets identified as the diagnosis worsened. Such observations were confirmed by the posthoc analysis. Although for the paper-based version, a difference emerged only between normal and MCI (*P*=.02), for the digitized test, the number of targets identified was significantly different between normal and dementia (*P*<.001) and normal and MCI (*P*<.001) groups.

#### Discriminative Power of Novel Indicators Derived From the Digitized Tests

The errors/targets ratio was significantly affected by the diagnosis only for the TMT-B (normal: 0.05 [IQR 0.05]; MCI: 0 [IQR 0.1]; dementia: 0.15 [IQR 0.39]; *X^2^*_2_=8.6; *P*=.01). The posthoc analysis revealed that the errors/targets ratio of the dementia group significantly differed from both the MCI (*P*=.01) and the normal (*P*=.04) groups.

ΔT significantly changed according to the diagnosis for all the 3 tests (TMT-A: normal 1.67 [IQR 0.78] seconds, MCI 2.10 [IQR 1.24] seconds, dementia 2.55 [IQR 0.48] seconds, *X^2^*_2_=12.2, *P*=.002; TMT-B: normal 3.66 [IQR 2.14] seconds, MCI 6.10 [IQR 7.45] seconds, dementia 10.07 [IQR 9.14] seconds, *X^2^*_2_=21.1, *P*<.001; and Bells Test: normal 4.46 [IQR 1.11] seconds, MCI 5.88 [IQR 2.15] seconds, dementia 6.59 [IQR 2.94] seconds; *X^2^*_2_=13.5, *P=*.001). The posthoc analysis reported that, for the 3 tests, ΔT of the dementia group was significantly greater than that of the normal group (TMT-A: *P*=.002; TMT-B: *P*<.001; Bells Test: *P*=.004). ΔT for the TMT-B and Bells Test showed significant differences between the normal and MCI groups (TMT-B: *P*=.003 and Bells Test: *P*=.004). Only for the TMT-B, ΔT significantly differed between the MCI and dementia groups (*P*=.03).

**Figure 5:**
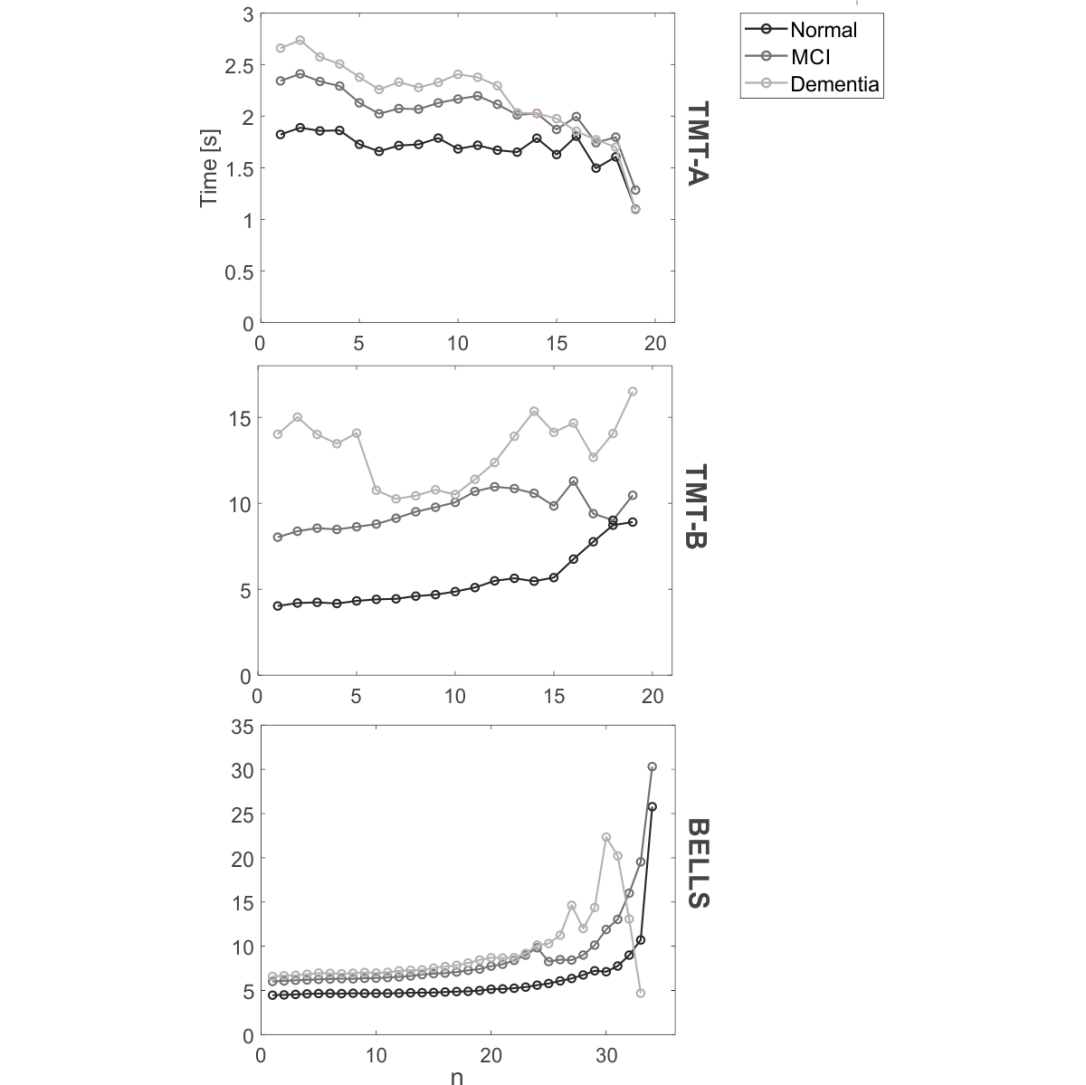
From top to bottom, ΔT_n for Trail Making Test (TMT)-Part A, TMT-B, and Bells Test. For each test, the 3 lines represent the average ΔT_n over the diagnosis groups (black: normal; dark gray: mild cognitive impairment; and light gray: dementia).

A qualitative analysis of ΔT_n was conducted and is presented in Figure 5. For all the 3 tests, the time between targets increases as the diagnosis worsens, as can be observed from the different offsets of the 3 lines. For TMT-A, the analysis revealed that ΔT_n decreased with test progression in all 3 patient groups. On the other hand, for all the groups, ΔT_n tended to increase toward the end of the TMT-B. As expected, for the Bells Test, the time between targets increased when identifying the final bells. In particular, a rapid increase can be observed after the 25th bell, especially in the dementia group. Note that the final drop of the dementia group curve is because of the fact that very few subjects were able to find more than 30 bells; therefore, the last points are obtained from the mean value over 1 or 2 patients and are not particularly representative of the entire dementia group.

**Figure 6:**
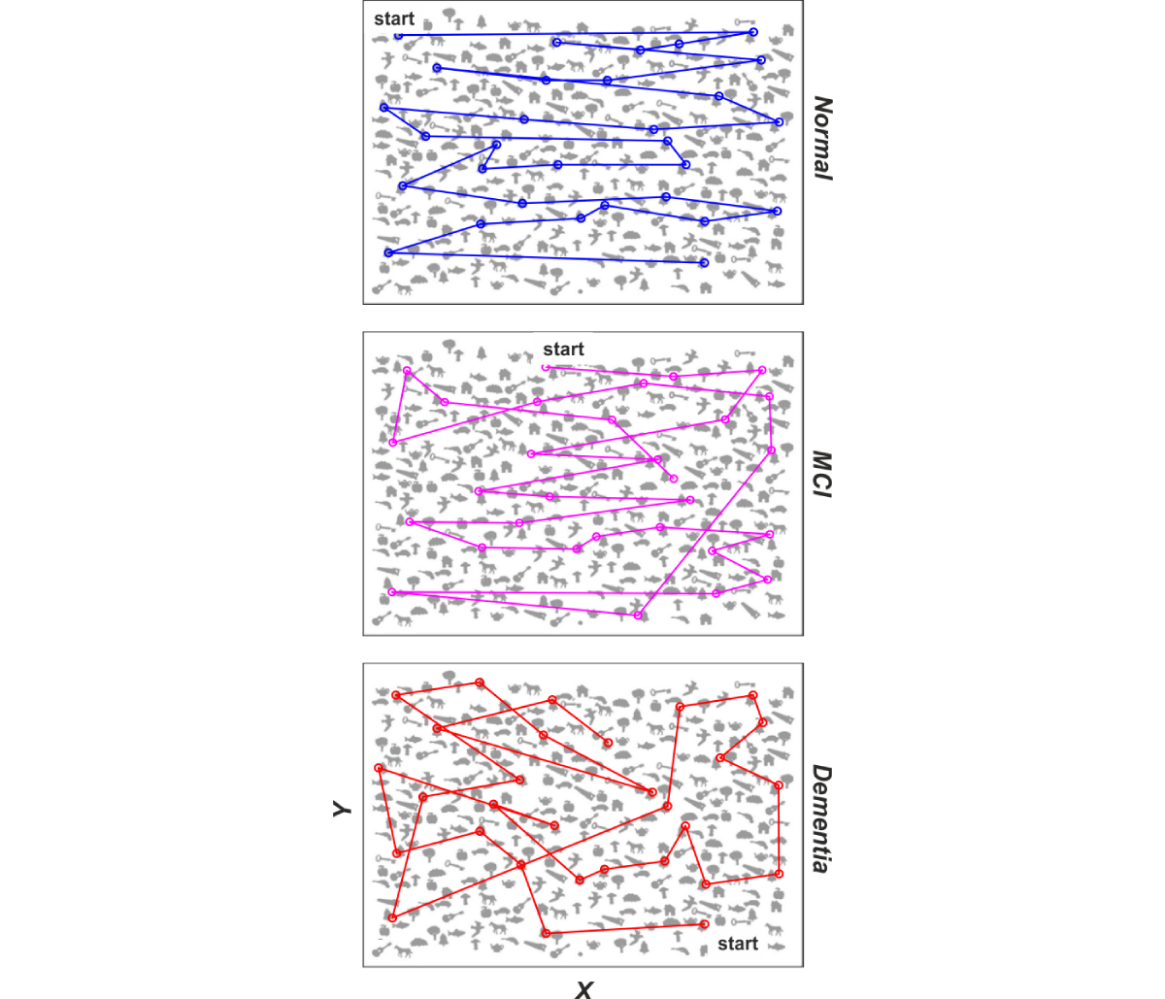
Examples of scanning strategies during the digitized Bells Test for 3 subjects, one for each diagnosis group; from top to bottom: normal (NIDmin=0), mild cognitive impairment (NIDmin=2), and dementia (NIDmin=4). MCI: mild cognitive impairment; NIDmin: minimum number of inversions in the direction.

Digitized testing is particularly suitable to gain insights into the subject’s visuo-attentional scanning during the Bells Test execution. Figure 6 reports representative examples of visuo-attentional strategies that emerged during the digitized Bells Test for the 3 diagnostic groups. As can be observed, the regularity of visuo-attentional scanning decreases with MCI and even more with the onset of dementia. This behavior is reflected in the NIDmin indicator, which presents an increasing trend with the worsening of the diagnosis, although not statistically significant (normal: 1 [IQR 1.5], MCI: 2 [IQR 3], and dementia: 3 [IQR 3.5]).

**Figure 7:**
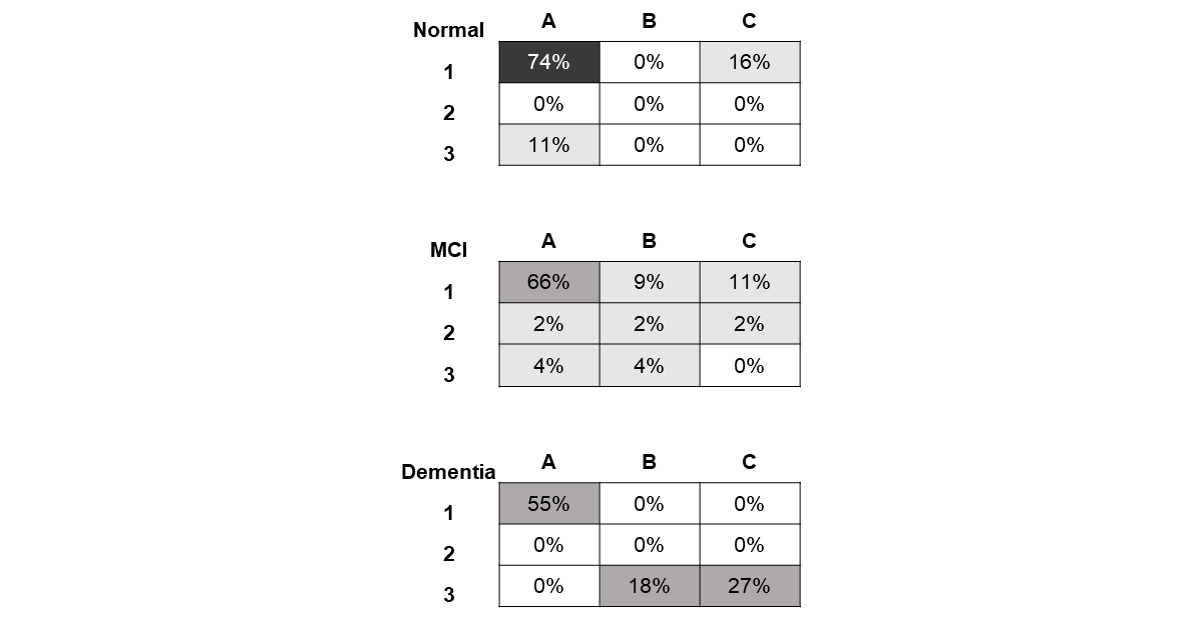
1Bell for the 3 diagnosis groups (from top to bottom: normal, mild cognitive impairment, and dementia). For each group, the table represents the writing area of the tablet divided into 9 subareas and the percentage of subjects who started the scanning strategy in each subarea (1Bell). MCI: mild cognitive impairment.

Another meaningful indicator that can be easily extracted and stored from the digitized Bells Test is related to the position of the first identified bell (1Bell), which is presented in Figure 7 (one panel for each diagnosis group). From Figure 7, it can be observed that although most of the subjects started the scanning strategy from the top-left corner (A1), this percentage decreases with the worsening of the diagnosis (normal: 74%, MCI: 66%, and dementia: 55%). In addition, the position of the first bell seems to be repeated among normal subjects, whereas an increased variability can be observed within the MCI group, for which the 1Bell indicator is spread over the canvas. In the dementia group, we noticed an additional reduction of the typical left-side start, although within-group variability decreases compared with MCI, as a significant percentage of patients with dementia (45% (5/12)) identified the first bell in the bottom-right corner (B3 and C3).

## Discussion

### Novelty of Our System

Following the information and communication technology (ICT) revolution, computer-based versions of most classical neuropsychological tests have been developed in both research [12,13,15,34] and commercial [35] domains. However, the full potential of ICT technology is not captured by these approaches as the digitized tests must be either administered under the supervision of a clinical expert or self-administered without proper instructions and without including a professional in the loop. With the goal of providing a monitoring tool that offers a reliable administration of the tests, we developed a platform offering a supervised digitized version of 2 neuropsychological tests commonly used to assess dementia: the TMT, in its A and B parts, and the Bells Test. Our supervised tool aims to support screening for the early onset of age-related cognitive decline.

Although, to the best of our knowledge, no previous work ever implemented a digitized version of the Bells Test, previous literature developed a computerized TMT [12,13]. However, the novelty of our system originates from the intent of deploying the platform in an uncontrolled environment for which supervision on the modality of use is needed to guarantee the validity of the recorded data. A VA was designed to mimic the supervision offered by a neuropsychologist during the administration of the paper-based version of the tests: it delivered instructions to the user, recognized the user’s answers, and produced the associated responses or interventions. The interaction between the VA and older adults is mediated through speech, as it is considered the most natural way of interacting with machines [27,36,37]. To this end, the dialogues were structured following the directions of clinical experts, and specific keywords and dictionaries were created to allow the VA to understand utterances with an equivalent semantic meaning. This way, the user can provide answers without constraints on the specific word, and the interaction is more robust and natural.

Together with web-based supervision, another key module of our system is the replay functionality, which was developed with the aim of allowing clinicians to remotely assess the test after execution. This functionality is crucial to increase the robustness and reliability of the system as it provides the clinician with the possibility of watching the test execution to better understand the obtained digitized score. Moreover, such functionality can be leveraged at clinical premises as the possibility of accessing test execution even at the end of the standard visit may potentially support the neuropsychologist in the diagnosis process, which could otherwise be hampered by the strict time schedule of the visits.

### Principal Findings

As a first step, we successfully conducted a laboratory-based functional testing of the developed platform to test the robustness of the VA and its integration with the TabletWebApp.

A necessary second step, before the deployment of the developed computerized testing in an uncontrolled environment, is the study of user acceptance and test validity, which are known to be the 2 main barriers to achieving feasible computerized assessment [17]. The results obtained from the sample of 83 older individuals were very promising. Indeed, in terms of usability, most users reported a favorable score, independent of their diagnosis (86% (19/22) normal, 77% (38/49) MCI, and 83% (10/12) dementia. Concerning user acceptance, the questionnaire reported strongly positive results for all the 3 diagnosis groups, with most users satisfied with the overall experience and appreciating the clarity of the graphical interface. This result is even more appreciable if we consider the overall low familiarity with the technology of the recruited subjects, especially with tablet devices. Our findings seem to confirm recent studies showing that although elders often portray themselves as digital illiterates, they show a surprising openness and ease of use when confronted with well-designed user-friendly technology [38]. In addition, it is worth pointing out that only 5 subjects refused to take part in the study, mostly because of exhaustion after the long neuropsychological visit, which took place right before the experimental session.

For system validity, we first leveraged the replay functionality to validate the software. For the Bells Test, the software was successfully validated, presenting a correlation coefficient between the 2 scores close to 1. In addition, for the TMT, satisfactory results were obtained in terms of specificity and accuracy, which were close to 100%. The sensitivity was slightly lower, mainly as some error-TMT events were misclassified as repetition-TMT when previously connected targets were hit again. Future work should focus on improving the sensitivity of the TMT software. Possible solutions may be the extension of the time-variant active area to all targets (and not restricted to the last one) and a postprocessing phase aimed at identifying possible misclassified error-TMT events.

We investigated both the concurrent and clinical validity of the computerized assessment compared with the traditional paper-and-pencil tests. For the TMT, the strong correlation between the 2 versions of the test suggests the potential of the digitized TMT to retain the same predictive power of the paper-and-pencil counterpart. This result confirms previous literature [13] for the digitized version of the TMT-B, whereas our results show a better outcome with regard to previous work on the TMT-A, for which only a moderate correlation was found between the digitized and paper-based versions. Concurrent validity is also supported by what emerged when investigating the effect of the neuropsychological diagnosis on the TMT score, with the time of completion increasing as the diagnosis worsened. Concerning the Bells Test, the weak correlation between the main outcome of the digitized and paper-based versions is somewhat surprising, especially given the fact that, contrary to the TMT, the layout of the digitized Bells Test was not modified with regard to the paper-based version, except for scaling down its layout size. Such differences in size are unlikely to explain the result as, owing to visual acuity, one would expect to identify a lower number of targets on a smaller support. However, in our case, there was no specific version of the test for which participants systematically achieved a better performance. A difference between the digitized and paper-based versions of the Bells Test also emerged when investigating the effect of the neuropsychological diagnosis on test scores. Although the result of the digitized test met our expectations, with the number of targets decreasing as dementia developed, the same trend was not found in the paper-based test, for which the number of bells identified by the dementia group was not statistically different from the normal group and was comparable with the MCI group. Thus, our results suggest a better discriminative power between mild and severe cognitive impairment for the digitized version of the Bells Test. Future work should investigate whether the reduced spatial contrast sensitivity that may characterize patients with dementia [39] could partially explain such a result.

One important advantage of computerized testing over traditional paper-based assessment is the possibility of quantifying additional information related to test execution strategies and not being restricted to the final test score. Such finer indicators may help clinicians gain insights into the patient’s executive functions. In this framework, our work extracted novel indicators that can capture information related to the between-target time and the user’s scanning strategy, which showed a decent discriminative power between diagnosis. In particular, the errors/targets ratio and ΔT indicators reported a very good between-diagnosis discriminant power, especially for the TMT-B; such a result is in line with previous literature on the paper-based TMT, which highlighted the effect of the diagnostic category only for TMT-B [40].

Depending on the test, different behaviors of ΔT_n indicator were observed. The TMT-A seemed to become easier toward the end of the execution (decrease in ΔT_n), and this trend appeared not to be affected by cognitive decline, as it was shared by all the 3 diagnostic groups. On the other hand, TMT-B test execution appeared to become more challenging for all the 3 groups when connecting the final targets, probably because of the mental fatigue induced by the test. In addition, for the Bells Test, the time between targets increased together with test progression, as expected.

In addition, the digitized version of the Bells Test allowed us to investigate the users’ scanning strategy. Although the scanning order can potentially be studied for the paper-based test also, the procedure of writing down the bell identification order is time-demanding for the clinician, resulting in its nonapplicability in real practice. On the other hand, digitized testing allows extraction and visualization of the patient’s scanning strategy in a convenient and easy way. As shown in Figure 7, the percentage of older adults starting the Bells Test scanning pattern from the top-left corner decreased with the severity of the diagnosis. Indeed, the normal group always started scanning from the sides, mostly from the left, as in the reading and writing processes. Instead, the MCI group showed the highest variability in the starting point of the scanning strategy. This variability decreased in the dementia group; however, almost half of the group identified the first bell in the bottom-right corner. An attempted explanation of what emerged is that, compared with healthy subjects, patients with MCI seem to be less attentive and present a mild early impairment of the planning strategy, reflecting slight executive function deficits [41]. In the dementia group, such deficits seem to be systematic, possibly revealing a visuo-attentional distortion.

### Future Direction

Given the promising results obtained from the fine digital-specific indicators, future work should strengthen the collaboration between technicians and clinicians, within fruitful bidirectional translational research, with the aim of defining novel indicators that could make digitized tests even more useful. However, it is crucial to keep in mind that such computational tools should not and cannot replace human care providers or clinicians, but they should provide possible recommendations for seeking further professional help, thus representing cost-effective solutions that are able to support the dementia screening programs of asymptomatic elders.

It is important to point out that, in this study, user acceptance and test validity were evaluated during an experimental protocol that did not include the VA. Indeed, digitized testing was performed at clinical premises under the supervision of a trained professional, without the guidance of the VA. Therefore, the reported results in terms of usability and concurrent validity do not apply to the full digitized testing platform; however, they represent a key and necessary step before the validation of the combined system. For this reason, we envisage that further usability and validity studies on the entire platform be performed in a home setting. To this end, the entire platform was deployed at the users’ home within the European MoveCare Project [42], and further usability results will be available at the end of the pilot project.

Our system requires a reliable internet connection. Future work should explore solutions that are able to work without a stable internet connection to increase the accessibility of such tools and foster their use in rural areas. A possible solution should leverage open-source technologies for the development of VAs that also work offline, pushing the execution of core system functionalities to the user’s side and introducing a local cache for data collection. In this way, we could loosen the persistent reliability of connectivity and allow the system to conduct all its functionalities under any network contingency. Indeed, functionalities running on the user’s side can also work offline, and locally cached data can be resynchronized as soon as the communication channel is restored.

Finally, future work should investigate the possibility of juxtaposing traditional and strategy-related indicators in the creation of a predictive model that can boost the classification process between neuropsychological diagnoses.

### Conclusions

Over the past decade, there has been a rapid evolution in the field of health screening methods to assist medical professionals to more accurately monitor older adults in relation to age-related conditions, such as dementia. With the goal of providing an effective tool to support the screening of cognitive decline, this work successfully designed and developed a supervised system for computerized neuropsychological assessment. The platform also includes a replay functionality designed to remotely inspect the user’s performance after test completion. Test supervision and replay functionalities potentially allow the delivery of the system directly at the user’s home or its use in clinical facilities without strict supervision to support the present medical services. Functional testing of the platform was successfully conducted. The digitized neuropsychological tests demonstrated very good concurrent validity, clinical validity, and a very high degree of user acceptance. The emerging positive results are necessary steps that pave the way toward the deployment of the system in clinical and nonclinical environments.

## Appendix

Multimedia Appendix 1 Supervised execution of the 3 tablet-based neuropsychological tests by an adult.

## Abbreviations

API: application programming interface
CAN-TAB: Cambridge Neuropsychological Test Automated Battery
CNSVS: CNS Vital Signs
GUI: graphical user interface
IRCCS: Istituti di Ricovero e Cura a Carattere Scientifico
MCI: mild cognitive impairment
MQTT: Message Queue Telemetry Transport
NID: number of inversions in the direction
NIDmin: minimum number of inversions in the direction
SUS: System Usability Scale
TMT: Trail Making Test
TMT-A: Trail Making Test-Part A
TMT-B: Trail Making Test-Part B
TMT-BA: Trail Making Test-Part B minus A
VA: vocal assistant
